# Astrocytes in Fear Memory Processing: Molecular Mechanisms Across the Amygdala–Hippocampus–Prefrontal Cortex Network

**DOI:** 10.3390/cells14181444

**Published:** 2025-09-16

**Authors:** Young-Rae Kim, Moonhyung Lee, Man S. Kim

**Affiliations:** 1 Translational-Transdisciplinary Research Center, Medical Science Research Institute, Kyung Hee University Hospital at Gangdong, College of Medicine, Kyung Hee University, Seoul 05278, Republic of Korea; cocoma317@khu.ac.kr; 2 Department of Medicine, Kyung Hee University College of Medicine, Seoul 02453, Republic of Korea; 3 Department of Gastroenterology and Hepatology, Kyung Hee University Hospital at Gangdong, Kyung Hee University College of Medicine, Seoul 05278, Republic of Korea; drmoon0101@khu.ac.kr

**Keywords:** astrocytes, fear memory, amygdala, hippocampus, prefrontal cortex, PTSD, gliotransmitters, astrocyte–neuron communication

## Abstract

Fear memory is a critical adaptive process that enables organisms to avoid potential threats and survive in complex environments. Traditionally viewed as a neuronal phenomenon, emerging evidence has demonstrated that astrocytes play a fundamental role in fear memory acquisition, consolidation, extinction, and retrieval across the distributed neural network encompassing the amygdala, hippocampus, and prefrontal cortex. This review presents recent advances in our understanding of the molecular mechanisms by which astrocytes modulate fear memory processing within the tripartite circuit. We examine how astrocytes contribute to synaptic plasticity, neurotransmitter regulation, metabolic support, and intercellular communication during the different phases of fear memory processing. Particular emphasis is placed on the region-specific functions of astrocytes, their dynamic interactions with neurons, and their therapeutic implications for treating fear-related disorders such as post-traumatic stress disorder (PTSD) and anxiety disorders. The integration of cutting-edge technologies, including spatial transcriptomics, optogenetics, and chemogenetics, has revealed sophisticated astrocyte–neuron communication mechanisms that challenge the traditional neuron-centric view of memory processing.

## 1. Introduction

Fear memory is one of the most evolutionarily conserved and critical learning processes, enabling organisms to rapidly acquire, store, and retrieve information regarding potentially threatening stimuli [1]. The neural circuitry underlying fear memory has been extensively characterized, with the amygdala, hippocampus, and prefrontal cortex forming a distributed network that orchestrates the acquisition, consolidation, and expression of fear memories [2,3,4]. However, our understanding of these processes has undergone a paradigm shift with the recognition that astrocytes, the most abundant glial cells in the brain, are not merely passive support cells, but are also active participants in information processing and memory formation [5,6].

Astrocytes form the “tripartite synapse” together with pre- and postsynaptic neuronal elements, where they can sense synaptic activity and respond by releasing gliotransmitters, modulating synaptic transmission, and providing metabolic support [7]. Recent technological advances, including cell-type-specific optogenetic and chemogenetic tools, spatial transcriptomics, and advanced imaging techniques, have revealed the sophisticated role of astrocytes in fear memory processing across different brain regions [8,9,10].

The amygdala–hippocampus–prefrontal cortex circuit exhibits remarkable functional specialization in fear memory processing. The amygdala, particularly the basolateral complex (BLA), is crucial for fear acquisition and the formation of fear associations [11]. The hippocampus contributes to contextual fear memory formation and extinction [12], while the prefrontal cortex, particularly the medial regions, regulates fear expression and extinction learning [13]. Within each of these regions, astrocytes display unique molecular signatures and functional properties that contribute to region-specific aspects of fear memory processing [9,12,13].

The amygdala–hippocampus–prefrontal cortex network operates through well-characterized anatomical and functional connections [14]. The BLA sends direct projections to the mPFC and receives reciprocal connections, forming a bidirectional circuit that regulates fear expression and extinction [14]. The hippocampus connects to both the amygdala and mPFC, providing contextual information that modulates fear responses [14]. During fear acquisition, the amygdala rapidly processes threat-related stimuli and sends signals to the mPFC and hippocampus [14]. Fear consolidation involves coordinated activity across all three regions, while extinction learning primarily engages mPFC-hippocampus interactions that inhibit amygdala responses [14]. This distributed processing allows for flexible, context-appropriate fear responses [14]. Within this network architecture, astrocytes in each region exhibit specialized functions that support the distinct computational roles of their respective brain areas, while also facilitating inter-regional communication through modulation of projection neurons and local circuit dynamics [5,15].

This review examines the current understanding of how astrocytes contribute to fear memory processing across the amygdala–hippocampus–prefrontal cortex network. We synthesize evidence from molecular, cellular, and behavioral studies to elucidate the mechanisms by which astrocytes modulate synaptic plasticity, neurotransmitter homeostasis, and neuronal excitability during fear learning. Furthermore, we discuss the therapeutic implications of targeting astrocytic function in the treatment of fear-related psychiatric disorders.

## 2. Astrocytes in the Amygdala: Orchestrating Fear Acquisition and Consolidation

### 2.1. Basolateral Amygdala Astrocytes in Fear Memory Formation

The basolateral amygdala (BLA) serves as the primary site of fear memory acquisition and consolidation, and astrocytes within this region play a pivotal role in these processes [16]. Recent spatial transcriptomic analyses have revealed that BLA astrocytes undergo profound transcriptional changes following fear conditioning, with memory-specific signatures that persist for weeks [8]. These transcriptional signatures implicate neuropeptide and BDNF signaling, MAPK and CREB activation, ubiquitination pathways, and synaptic connectivity as key components of long-term memory formation [8].

Chemogenetic studies targeting astrocytes, specifically in the basolateral amygdala (BLA), have shown that these glial cells play a crucial role in the formation of fear memories [9]. When Gq-coupled receptors in BLA astrocytes are activated during fear learning, they enhance the formation of auditory-cue-associated fear memories, while leaving other less stressful memory processes and anxiety-related behaviors unaffected [9]. This effect occurs only during the memory acquisition phase, and astrocytic activation during memory recall does not influence fear memory expression [9]. The underlying mechanism involves the regulation of neural communication between the amygdala and prefrontal cortex [9,17]. When activated, astrocytes boost the activity of BLA neurons that project to the medial prefrontal cortex (mPFC) and improve the synchronization of neural oscillations between the BLA and mPFC regions [9].

Recent optogenetic research has provided deeper insights into the precise timing of astrocytic contributions to fear memory. Astrocytes in the amygdala demonstrate context-dependent influences on memory establishment, where optical stimulation via channelrhodopsin-2 (ChR2) enhances both fear detection and initial memory encoding, while paradoxically hindering the consolidation process [10]. In contrast, optical inhibition through archaerhodopsin-T (ArchT) disrupts memory acquisition during strong aversive experiences and protects established fear memories from deteriorating over time [10]. These observations highlight the intricate, time-sensitive nature of astrocytes in fear memory mechanisms.

### 2.2. Molecular Mechanisms of Amygdala Astrocyte Function

The molecular mechanisms underlying astrocytic modulation of fear memory in the amygdala involve multiple signaling pathways and cellular processes.

**Figure 1:**
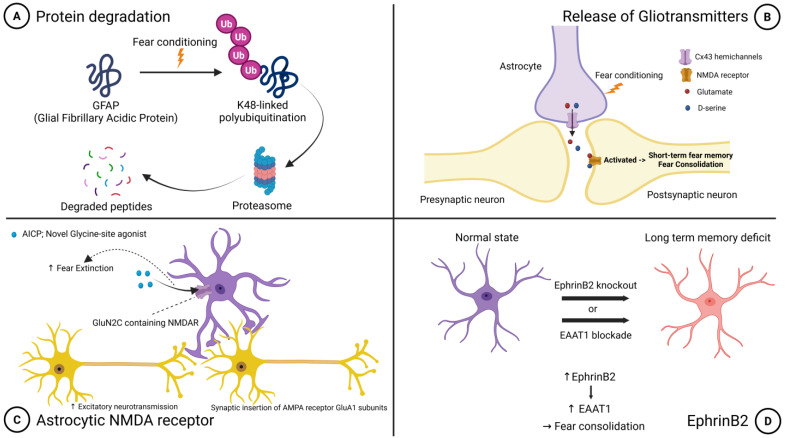
Molecular Mechanisms of Basolateral Amygdala Astrocyte Function in Fear Memory Formation. Four key molecular pathways by which astrocytes in the basolateral amygdala regulate fear memory processes. (**A**) Protein degradation pathway: Fear conditioning triggers K48-linked polyubiquitination of GFAP (Glial Fibrillary Acidic Protein), leading to proteasomal degradation essential for fear memory consolidation. (**B**) Release of gliotransmitters: Cx43 hemichannels in astrocytes release glutamate and D-serine during fear conditioning, which activate NMDA receptors on adjacent neurons to facilitate short-term fear memory formation and consolidation. (**C**) Astrocytic NMDA receptor pathway: Astrocytic NMDA receptors containing the GluN2C subunit can be activated by the novel glycine-site agonist AICP, leading to enhanced fear extinction through presynaptic facilitation of excitatory neurotransmission and synaptic insertion of AMPA receptor GluA1 subunits. (**D**) EphrinB2-EAAT1 pathway: EphrinB2 signaling pathway regulates long-term fear memory consolidation through control of astrocytic glutamate uptake. Under normal conditions (left), EphrinB2 signaling promotes EAAT1 expression, enabling efficient glutamate clearance and supporting memory consolidation. EphrinB2 knockout or EAAT1 pharmacological blockade (right) disrupts this pathway, leading to impaired glutamate uptake and long-term memory deficits. The molecular pathway demonstrates how EphrinB2 → EAAT1 signaling in basolateral amygdala astrocytes is essential for fear memory consolidation, illustrating the active regulatory role of astrocytes in memory formation rather than passive support function. Figure created with BioRender.com.

Protein degradation mechanisms play a fundamental role in memory processes, as both neuronal and astrocytic protein degradation have been shown to be essential for fear memory consolidation [18]. Proteomic studies have identified GFAP, an essential astrocytic structural protein, as a K48 polyubiquitination target in both sexes following fear conditioning, confirming that astrocytes undergo protein degradation during fear memory formation [19] (Figure 1A and Table 1).

Additionally, the release of gliotransmitters via connexin 43 (Cx43) hemichannels constitutes another essential mechanism by which astrocytes in the amygdala regulate fear memory processes [20]. Fear conditioning enhances Cx43 hemichannel function in BLA astrocytes, and selective blockade of these hemichannels induces memory deficits without affecting learning [20]. These memory deficits can be rescued by the simultaneous administration of glutamate and d-serine, implicating NMDA receptor involvement. Notably, D-serine passes through the Cx43 hemichannels and, in combination with glutamate, is required for proper NMDAR function during conditioning [20] (Figure 1B).

The involvement of astrocytic NMDA receptors in fear extinction has been demonstrated in studies using the novel glycine-site agonist, AICP [21]. Astrocytic NMDA receptors containing the GluN2C subunit in the BLA facilitate fear extinction, and chemogenetic activation of BLA astrocytes promotes fear extinction [21]. This mechanism involves the presynaptic facilitation of excitatory neurotransmission and synaptic insertion of AMPA receptor GluA1 subunits [21] (Figure 1C).

EphrinB2 has emerged as a key regulator of fear memory consolidation in BLA astrocytes [21]. Astrocyte-specific ephrinB2 knockout in the BLA selectively disrupts long-term memory formation while leaving short-term memory intact [22]. This effect is mediated by ephrinB2’s control of excitatory amino acid transporter 1 (EAAT1) expression in astrocytes, as ephrinB2 loss leads to reduced EAAT1 levels [22]. Consistent with this mechanism, pharmacological blockade of EAAT1 using specific inhibitors during fear conditioning produces similar long-term memory deficits, demonstrating that ephrinB2 regulates memory consolidation by controlling astrocytic glutamate uptake [22] (Figure 1D).

## 3. Hippocampal Astrocytes: Guardians of Context and Extinction

### 3.1. Functional Roles of Hippocampal mPFC in Fear Memory

The hippocampus, particularly the CA1 region, plays a crucial role in the formation and extinction [12,39]. A recent study has revealed that hippocampal CA1 astrocytes exhibit de novo calcium dynamics during fear extinction and play a critical role in this process [23]. Suppression of astrocytic calcium activity impairs fear extinction, whereas enhancement promotes it [23]. This regulatory mechanism depends on cholinergic projections from the posterior basal forebrain (pBF) that stimulate CA1 astrocytic calcium responses via α4 and α7 nicotinic acetylcholine receptors [23]. The clinical significance of this pathway is highlighted by evidence that donepezil, an acetylcholinesterase inhibitor in therapeutic use, increases CA1 astrocytic calcium dynamics and promotes fear extinction [23]. This neuron-astrocyte signaling pathway is a potential therapeutic target for fear- and anxiety-related disorders [23].

Recent groundbreaking studies have established that hippocampal astrocytes form learning-associated ensembles that actively participate in memory formation, consolidation, and recall in various memory paradigms. In the hippocampus, learning events activate specific subsets of astrocytes that exhibit experience-dependent plasticity through enhanced c-Fos expression, altered calcium signaling patterns, and unique transcriptional profiles distinguished by factors such as nuclear factor I-A (NFIA) [24]. Importantly, these learning-associated astrocyte (LAA) ensembles demonstrate both anatomical and functional coupling with neuronal engrams, forming clustered networks around engram neurons and synaptic sites [24]. The functional significance of these astrocyte ensembles has been demonstrated through selective manipulation studies, showing that their activation is necessary for normal memory recall and is sufficient to trigger memory-associated behaviors, even in neutral contexts [24].

### 3.2. Molecular Mechanisms of Hippocampal Astrocyte Function

The molecular mechanisms by which hippocampal astrocytes regulate fear memory processes encompass several key pathways, including purinergic signaling and protein synthesis regulation.

**Figure 2:**
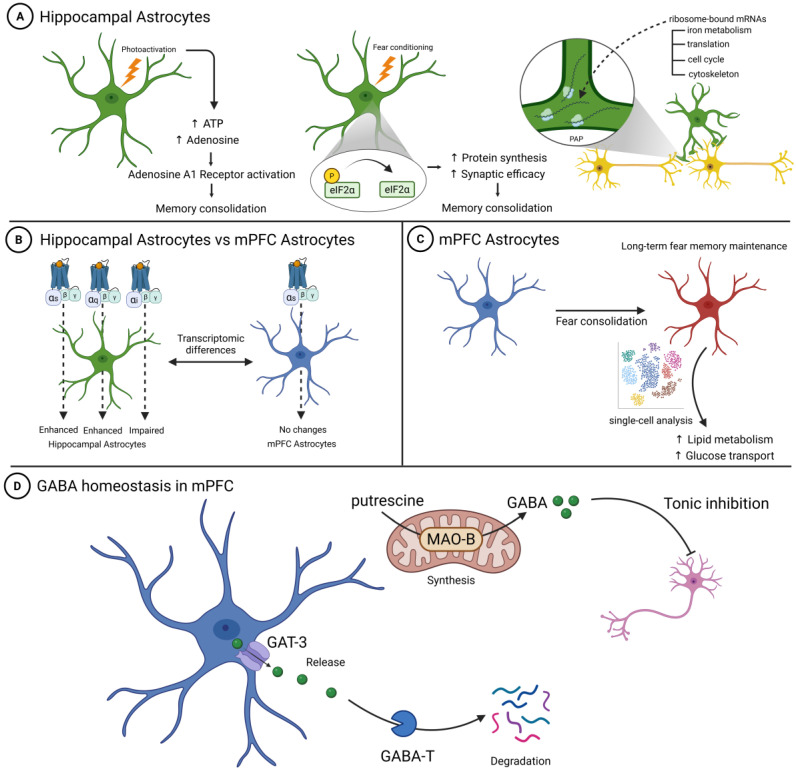
Regional Differences in Astrocytic Contributions to Fear Memory Processing. Comparison of molecular mechanisms and functional responses between hippocampal and medial prefrontal cortex astrocytes in fear memory formation. (**A**) Hippocampal astrocyte mechanisms: Three key pathways regulate fear memory in the hippocampus. Left: Optogenetic photoactivation triggers ATP release and conversion to adenosine, leading to A1 receptor activation and memory consolidation modulation. Middle: Fear conditioning induces eIF2α dephosphorylation, enhancing protein synthesis and synaptic efficacy for memory consolidation. Right: Perisynaptic astrocytic processes (PAPs) contain ribosome-bound mRNAs encoding proteins for iron metabolism, translation, cell cycle, and cytoskeleton functions, supporting local protein synthesis during learning. (**B**) Regional differences in DREADD responses: Comparison of astrocytic G-protein coupled receptor (GPCR) signaling effects between hippocampal and mPFC astrocytes. Hippocampal astrocytes show enhanced responses to Gs and Gq activation and impaired responses to Gi activation, while mPFC astrocytes show no significant changes across all GPCR subtypes, reflecting distinct transcriptomic profiles between brain regions. (**C**) mPFC astrocyte metabolic reprogramming: Single-cell transcriptomic analysis reveals persistent metabolic changes in mPFC astrocytes during remote fear memory consolidation. Fear conditioning triggers sustained upregulation of genes involved in lipid metabolism and glucose transport, leading to enhanced energetic support for long-term memory maintenance. The transition from baseline (blue) to metabolically active state (red) demonstrates how mPFC astrocytes maintain an altered functional state that supports remote memory storage. (**D**) GABA homeostasis in mPFC: Astrocytic regulation of ambient GABA levels through three interconnected pathways. MAO-B-dependent synthesis converts putrescine to GABA in astrocytic mitochondria, GAT-3 transporters mediate GABA release into extracellular space, and GABA-T enzyme controls GABA degradation. This balanced homeostatic system enables astrocytes to modulate tonic inhibition and fear extinction processes through precise control of ambient GABA levels. Figure created with BioRender.com.

Hippocampal astrocytes modulate fear memory through purinergic signaling. Optogenetic stimulation of hippocampal astrocytes impairs memory consolidation and produces lasting reductions in contextual fear memory, along with diminished fear-related anxiety behaviors [12]. In vivo microdialysis studies have revealed that astrocyte photoactivation elevates extracellular ATP and adenosine levels [12]. Intracerebral blockade of adenosine A1 receptors reverses the reduction in fear memory, whereas A1 receptor agonist treatment replicates the effects of astrocyte stimulation [12]. This adenosine-dependent mechanism offers a promising therapeutic approach for pathological fear-related disorders because it selectively interferes with the consolidation phase of memory processing without disrupting acquisition or retrieval [12] (Figure 2A).

Astrocyte-mediated regulation of protein synthesis is another important mechanism underlying fear memory in the hippocampus [25]. During contextual fear conditioning, hippocampal astrocytes undergo dynamic changes in eIF2α phosphorylation states, with reduced phosphorylation levels facilitating enhanced protein production [25]. Genetically decreased eIF2α phosphorylation specifically in hippocampal astrocytes results in improved performance in both contextual and spatial memory tasks, while simultaneously lowering the threshold required for inducing persistent synaptic modifications [25]. These findings indicate that fear learning triggers eIF2α dephosphorylation in astrocytes, which subsequently strengthens synaptic efficacy and supports the consolidation of hippocampal-dependent memories through alterations in synaptic communication [25] (Figure 2A).

Additionally, local protein synthesis occurs during perisynaptic astrocytic processes (PAPs) [26]. Hippocampal PAPs contain differentially distributed ribosome-bound mRNAs that encode proteins that regulate iron metabolism, translation, cell cycle, and cytoskeleton functions [27]. These findings show that local protein synthesis in astrocytes plays a role in memory formation and learning processes [27] (Figure 2A).

## 4. Prefrontal Cortex Astrocytes: Executives of Fear Regulation

### 4.1. Functional Roles of Hippocampal Astrocytes in Fear Memory

The medial prefrontal cortex (mPFC) plays a crucial role in fear memory regulation, extinction, and cognitive control of fear responses. Astrocytes in the mPFC contribute to these functions via multiple mechanisms [28,29].

Astrocytic glucocorticoid signaling in the prefrontal cortex plays an important role in fear memory processes [13]. Selective knockout of glucocorticoid receptors in the mPFC astrocytes enhances freezing responses during fear recall. Notably, extinction learning remains intact, but female mice specifically exhibited deficits in recalling extinction memories, suggesting sex-dependent roles for astrocytic glucocorticoid receptors in fear memory regulation [13].

During fear conditioning, mPFC astrocytes exhibit dynamic calcium responses to fear-relevant stimuli such as foot shock and predator odors [29]. These astrocytes show differential activation patterns depending on stimulus predictability, with unpredictable aversive stimuli inducing more intense and rapid calcium responses than predictable stimuli do [29]. This suggests that mPFC astrocytes may contribute to fear memory encoding by detecting and responding to the salience and unpredictability of threatening stimuli [29].

### 4.2. Molecular Mechanisms of mPFC Astrocytes

mPFC astrocytes employ diverse molecular mechanisms to modulate fear memory processing. Chemogenetic activation of astrocytic Gs signaling during contextual fear memory encoding and consolidation did not significantly alter remote memory expression, nor did it affect the size or reactivation patterns of cortical engram neuron populations [28]. This contrasts with findings in hippocampal astrocytes, where Gq- and Gi-DREADD manipulations enhanced or impaired fear memory, respectively, suggesting region-specific roles for different astrocytic GPCR subtypes in memory processing [28].

The lack of an effect of Gs pathway activation on cortical engram function may reflect the distinct transcriptome profiles between cortical and hippocampal astrocytes, potentially leading to different downstream molecular responses to GPCR activation [28,30]. While Gs activation typically increases cAMP production and promotes lactate shuttling mechanisms that support recent memory retention in the hippocampus, these pathways appear to be insufficient to alter persistent cortical memory engrams [28] (Figure 2B).

Single-cell transcriptomic analysis revealed that mPFC astrocytes undergo persistent metabolic reprogramming during remote fear memory consolidation, with sustained upregulation of genes involved in lipid metabolism and glucose transport [31]. This metabolic remodeling suggests that prefrontal astrocytes provide the ongoing energetic support necessary for long-term fear memory maintenance, indicating that mPFC astrocytes maintain an altered functional state that supports remote memory storage (Figure 2C).

#### Astrocytic GABA Synthesis and Fear Extinction

Astrocytic GABA synthesis and release represent a fundamental mechanism by which glial cells modulate fear extinction in the medial prefrontal cortex. Unlike conventional neuronal GABAergic transmission that occurs at synaptic sites, astrocytes contribute to ambient GABA levels through extrasynaptic release mechanisms that mediate tonic inhibition [32]. This tonic GABAergic regulation is particularly important for fear extinction processes, as it modulates the baseline excitability of cortical neurons involved in extinction learning [33].

The molecular pathway underlying astrocytic GABA synthesis involves the degradation of putrescine by monoamine oxidase B (MAO-B) enzymes localized in astrocytic mitochondria [34]. In reactive astrocytes, this pathway becomes dysregulated, leading to excessive GABA production and release [35]. Studies using repetitive pulsed-wave ultrasound stimulation have demonstrated that astrocytic activation directly modulates ambient GABA levels, resulting in sustained neural inhibition [36], confirming that astrocytes can dynamically regulate GABA tone in response to external stimuli.

In addition to synthesis pathways, transporters and degradative enzymes also play a key role in regulating astrocytic GABA homeostasis. For instance, astrocytic GAT-3-mediated GABA release has been shown to tonically activate GABAB receptors, reducing network activity in developing cortical circuits [37]. Complementing this, the sole GABA-degrading enzyme, GABA transaminase (GABA-T), is also critical. Suppression of astrocytic GABA-T enhances tonic inhibition but impairs spatial memory formation, demonstrating that balanced GABA metabolism is critical for cognitive function [38] (Figure 2D).

Taken together, these findings clearly show that astrocytes precisely control ambient GABA levels through multiple pathways, including MAO-B-dependent synthesis, GAT-3-mediated release, and GABA-T-dependent degradation. Considering that this regulation directly impacts functions like developmental excitability and spatial memory, it is a plausible inference that these mechanisms also underlie higher cognitive processes such as fear extinction. Therefore, modulating these astrocytic pathways represents a promising therapeutic strategy for treating disorders characterized by impaired fear extinction.

## 5. Molecular Mechanisms of Astrocyte–Neuron Communication in Fear Memory

While the previous sections focused on the specialized functions of astrocytes within individual brain regions—detailing how astrocytes contribute uniquely to fear acquisition (amygdala), contextual processing (hippocampus), and executive control (prefrontal cortex)—this section examines the fundamental molecular mechanisms that underlie astrocyte–neuron communication across the entire fear memory network. Rather than representing repetition of regional functions, this section synthesizes the common molecular principles that enable astrocytes to support fear memory processing at the network level. These mechanisms-including gliotransmitter systems, calcium signaling, structural plasticity, and transcriptional regulation-operate across all brain regions but manifest differently based on regional specialization. Understanding these core mechanisms is essential for comprehending how astrocytes coordinate fear memory functions across the distributed neural network and how disruption of these processes leads to fear-related pathologies.

### 5.1. Gliotransmitter Systems

Astrocytes communicate with neurons by releasing various gliotransmitters, including glutamate, GABA, ATP, adenosine, and D-serine [7,15]. In the context of fear memory formation, these gliotransmitters perform distinct functions in different brain regions. In the amygdala, astrocytic release of glutamate and D-serine through Cx43 hemichannels is essential for NMDA receptor (NMDAR) activation during fear conditioning [20]. In the hippocampus, the astrocytic release of ATP and its conversion to adenosine modulate fear memory consolidation through A1 receptor activation [12].

### 5.2. Calcium Signaling and Astrocyte Activation

Astrocytic calcium signaling serves as a critical mediator of astrocyte–neuron communication. In the amygdala, astrocyte hypoactivity, characterized by decreased frequency, amplitude, and size of calcium events, directly affects neuronal function following early life stress [40]. The bidirectional nature of this communication is evidenced by the finding that astrocytic dysfunction alone enhances neuronal excitability, suggesting that astrocytes tune engram formation and memory specificity [40]. Hippocampal astrocytes also utilize calcium signaling to communicate with neurons, where cholinergic inputs drive astrocytic calcium dynamics essential for fear extinction [23]. Specifically, posterior basal forebrain cholinergic signaling directly activates hippocampal CA1 astrocytes through α4 and α7 nicotinic acetylcholine receptors, demonstrating that neuromodulatory inputs can selectively target astrocytic networks independent of local neuronal activity [23]. This astrocyte–neuron communication represents a bidirectional process in which neuronal activity can modulate astrocytic calcium responses, and astrocytic dysfunction can alter neuronal excitability and memory formation.

### 5.3. Structural Plasticity and Synaptic Remodeling

Astrocytes undergo dynamic structural plasticity in response to fear learning, which critically modulates synaptic function and memory formation. During fear memory consolidation, perisynaptic astrocytic processes transiently retract from hippocampal synapses, facilitating glutamate spillover and enhancing NMDAR activation [41]. This experience-induced withdrawal of astrocytic coverage from the synaptic cleft represents a temporally regulated mechanism that gates fear memories [41]. In the lateral amygdala, threat conditioning results in a transient increase in the density of synapses lacking astrocytic contact, particularly in enlarged spines containing polyribosomes and the spine apparatus, whereas synapses with astrocytic contacts become smaller after conditioned inhibition [42]. This suggests that astrocytic processes are absent during synapse enlargement but present during synapse shrinkage, with contact with astrocytic processes opposing synapse growth during memory consolidation [42]. The structural remodeling of astrocytic processes is mediated by actin-binding proteins, such as ezrin, which regulate process morphology and synaptic contact [41]. Additionally, Rac-1 activity in basolateral amygdala astrocytes modulates fear memory formation through its effects on astrocyte structural changes [6].

### 5.4. Transcriptional Regulation

Transcriptomic profiling of learning-associated astrocyte (LAA) ensembles revealed unique molecular signatures that distinguished them from non-activated astrocytes [24]. A particularly critical finding was the upregulation of nuclear factor I-A (NFIA), a transcription factor whose expression is necessary and sufficient for memory recall [24]. Selective deletion of NFIA in LAAs impairs memory recall without affecting other hippocampus-dependent behaviors, underscoring the specificity of this transcriptional program [24].

These ensembles also upregulate synapse-related genes and known NFIA targets [24], indicating that astrocytic transcriptional responses may directly enhance synaptic function and astrocyte–neuron communication. Unlike immediate early gene activation in neurons, these transcriptional changes in astrocytes represent a durable, experience-dependent transcriptional plasticity uniquely tailored to support long-term memory encoding through glial-neuronal interactions [24].

In addition to transcription factor–mediated regulation, post-translational modifications of the transcriptional machinery also contribute to astrocyte–neuron communication during fear memory formation. Among these, O-GlcNAcylation in astrocytes modulates gene expression programs involved in neurotransmitter transport and ion channel regulation, which are key processes in astrocyte–neuron interactions [43]. The knockout of astrocytic O-GlcNAc transferase (OGT) results in elevated GFAP expression and increased transcription of genes related to potassium- and voltage-gated ion channel activity, suggesting enhanced astrocytic responsiveness and synaptic communication during fear memory formation [43].

## 6. Modulating Factors of Astrocytic Function in Fear Memory

### 6.1. Early-Life Stress and Astrocyte Dysfunction

Early life stress leads to long-lasting astrocytic dysfunction in the amygdala, characterized by compromised synaptic function and increased neuronal activity [40]. When researchers experimentally reduced astrocytic calcium signaling or disrupted astrocytic networks in the amygdala, they observed similar fear generalization patterns, suggesting that astrocytic dysfunction is a key mechanism underlying stress-induced behavioral changes [40] (Table 2).

Recent longitudinal studies have revealed that early life stress affects hippocampal plasticity through long-term alterations in astrocytic function. As part of the tripartite synapse, astrocytes are critical modulators of neuronal plasticity and are well-positioned to mediate the long-term effects of early life stress on neuronal circuits [44]. These stress-induced astrocytic changes include altered neurotransmitter transporter expression and gap junction connectivity, which may underlie the greater vulnerability to mood and anxiety disorders later in life [45,46,47].

### 6.2. Trauma-Related Astrocyte Changes and Neuroinflammation

Traumatic brain injury alters gene expression profiles across multiple amygdala cell populations, with notable changes observed in astrocytic transcriptomes [48]. Among the upregulated genes, decorin has emerged as a key mediator of trauma-induced fear responses, as knockout of decorin in the amygdala reduces trauma-associated fear conditioning [48]. The underlying mechanism involves the influence of decorin on perineuronal net integrity, ultimately disrupting the glutamate-GABA balance within the amygdala circuits [48].

Studies using rodent models of PTSD revealed that glial cells undergo stress-induced molecular alterations in response to therapeutic interventions [49]. Ketamine administration can reverse pathological changes in astrocytes and microglia, normalizing key cellular markers, including S100B protein levels, neurotrophic factors, such as BDNF and FGF2, and synaptic structural proteins [49]. These observations demonstrate that glial reactivity and the associated neuronal dysfunction are fundamental factors that determine an individual’s susceptibility to trauma-related disorders.

Comorbid traumatic brain injury and PTSD produce more severe deficits than either condition alone, with increased neuroinflammation being the key mechanism. Studies have shown that the order of experiencing TBI and PTSD is a major determinant of the outcome, with chronic variable stress followed by closed-head injury producing the greatest cognitive deficits and hippocampal inflammation [50]. Notably, this inflammatory response often involves complex interactions between resident brain cells and the peripheral immune system, highlighting a potential link between central and systemic processes. [51]

### 6.3. Sex Differences in Astrocytic Contributions to Fear Memory

Emerging evidence has revealed significant sex differences in the astrocytic contribution to fear memory formation [13,19]. Proteomic analysis of the amygdala following fear conditioning showed that males and females differ in the proteins targeted for degradation, with only three proteins overlapping between the sexes [19]. In females, proteins involved in vesicle transport and microtubule function were preferentially targeted, whereas in males, proteins involved in the cytoskeleton, ATP synthesis, and cell signaling were altered abundance [19].

Astrocyte calcium events exhibit sexual dimorphism, with a greater amplitude and size observed in female mice, which may explain the more profound fear discrimination impairments in females following astrocyte manipulation [40]. Stress exposure eliminates these sex differences and induces homogeneous calcium signatures between sexes [40].

Sex differences are also evident in astrocytic glucocorticoid receptor function, with female mice showing specific impairments in extinction memory recall when glucocorticoid receptors are knocked out in prefrontal cortex astrocytes [13]. These findings have important implications for understanding sex differences in PTSD susceptibility and treatment responses [13].

Brain-derived neurotrophic factor (BDNF), which is closely linked to astrocytic function, also shows sex-specific patterns in chronic pain. In non-depressed participants, women with severe pain showed lower BDNF concentrations than those without chronic pain, whereas in men with depression, both severe and interfering pain were linked to lower BDNF levels [44]. These findings suggest that the relationship between BDNF and pain processing is distinctly modulated by sex and mental health status, which may extend to fear-related disorders.

### 6.4. Clinical Evidence of Astrocytic Dysfunction in Fear-Related Disorders

Clinical studies have provided compelling evidence for astrocytic dysfunction in human subjects with fear-related disorders, supporting the translational relevance of preclinical findings. Blood-based biomarkers of astrocytic activation have revealed complex patterns of glial dysfunction in trauma-exposed populations.

A cross-sectional study of 1520 World Trade Center responders demonstrated that severe PTSD symptoms were associated with reduced plasma GFAP levels, suggesting glial suppression rather than activation [52]. Similarly, the TRACTS study of 550 post-9/11 veterans found that blast exposure was associated with lower GFAP levels and more severe psychological symptoms [53]. In contrast, the ADVANCE-TBI study of UK military personnel found elevated plasma GFAP levels 8 years post-injury, with higher levels correlating with unemployment and poor functional outcomes [54].

Sex differences in astrocytic responses have been consistently observed. Female TBI patients exhibited significantly higher GFAP and tau levels compared to males, along with more severe symptoms [55]. Furthermore, this glial dysfunction is often exacerbated by common comorbidities like chronic sleep disruption, a well-established factor in trauma-exposed individuals [56,57], which itself is known to directly impact astrocyte homeostasis [58,59].

## 7. Therapeutic Implications and Future Directions

### 7.1. Targeting Astrocytic Pathways for PTSD Treatment

The identification of specific astrocytic mechanisms involved in fear memory processing opens new therapeutic avenues for treating PTSD and related disorders. The cholinergic pathway from the basal forebrain to hippocampal astrocytes represents a particularly promising target, because clinically available acetylcholinesterase inhibitors can enhance astrocytic calcium dynamics and facilitate fear extinction [23] (Table 3).

Adenosine A1 receptor agonists represent another potential therapeutic approach, as they can mimic the memory-dampening effects of astrocyte activation in the hippocampus [10]. This strategy could be particularly valuable for preventing the consolidation of traumatic memories when administered shortly after trauma exposure [12].

Building on the astrocytic GABA mechanisms detailed in Section Astrocytic GABA Synthesis and Fear Extinction, the reversible MAO-B inhibitor KDS2010 has shown promise in restoring astrocytic GABA homeostasis and improving fear extinction in PTSD models [32]. This compound has completed Phase I clinical trials and represents a targeted approach to correcting the astrocytic dysfunction underlying PTSD symptomatology [32].

### 7.2. Precision Medicine Approaches and Personalized Treatment

The recognition of sex differences in astrocytic contributions to fear memory suggests that precision medicine approaches may be necessary for the optimal treatment of fear-related disorders [13,19]. Female-specific alterations in astrocytic glucocorticoid receptor function may require different therapeutic strategies than those used in males [13]. This sex-specific difference in stress hormone responsiveness could inform the development of targeted hormonal interventions for the treatment of PTSD, particularly considering the high prevalence of PTSD among women.

Single-cell transcriptomic approaches have revealed the molecular signatures of astrocytic dysfunction in fear-related disorders, potentially enabling the identification of specific transcriptional biomarkers that can guide personalized therapeutic interventions targeting astrocytic metabolic pathways [31].

### 7.3. Targeting Neuroimmune Pathways in Fear-Related Disorders

Recent studies have identified neuroimmune interactions as potential therapeutic targets for fear-related disorders. Chronic stress recruits inflammatory monocytes to the brain meninges, which modulate amygdala astrocyte function through the EGFR-PTPRS signaling pathway, ultimately affecting neuronal NR2F2 expression and fear behavior [51]. This mechanism has been validated in patients with MDD with similar molecular signatures. In preclinical studies, psychedelic compounds such as psilocybin and MDMA reversed meningeal monocyte accumulation and restored normal astrocyte–neuron communication [51]. These findings expand the therapeutic landscape beyond traditional neurotransmitter-focused approaches, suggesting that targeting peripheral immune-brain interactions may provide novel treatment strategies for PTSD and anxiety disorders. This neuroimmune perspective complements existing astrocyte-targeted therapies and opens up new possibilities for combination treatments that address both central neural circuits and peripheral inflammatory processes.

### 7.4. Astrocyte Heterogeneity and Fear Memory Specificity

Computational analyses of astrocyte transcriptomic data revealed distinct modules associated with neurological diseases, including stress-responsive and extracellular matrix modules, which may be particularly relevant to fear memory formation [60]. The identification of A1 (neurotoxic) and A2 (neuroprotective) reactive astrocyte states provides a framework for understanding how different astrocytic populations contribute to adaptive fear learning or pathological fear generalization [61,62]. However, a recent consensus suggests moving beyond the binary A1/A2 classifications to more nuanced multivariate approaches that capture the complex functional heterogeneity of reactive astrocytes [62].

Single-cell studies have demonstrated that astrocyte subtypes exhibit distinct spatiotemporal responses to pathological stimuli, with white matter astrocytes showing unique proliferative capacities and molecular signatures compared to gray matter astrocytes [63]. The recognition of astrocyte heterogeneity opens new possibilities for precision glial therapy in fear-related disorders, rather than targeting all astrocytes.

### 7.5. Astrocyte-Sleep-Fear Memory Axis

Studies of sleep deprivation have revealed that astrocytes undergo profound changes [58]. Sleep-deprived individuals exhibit reduced serum levels of astrocytic markers, including AQP4, connexin-30, and connexin-43, suggesting that astrocyte dysfunction is linked to compromised sleep quality and cognitive function [59]. Disruption of astrocytic gap junction coupling impairs energy substrate delivery, and compromised lactate outflow can hamper long-term neuronal plasticity and energy metabolism [58].

These findings provide a mechanistic explanation for the well-established clinical observation that sleep disturbance exacerbates PTSD symptoms and impairs fear extinction learning [56,57]. Sleep disruption prior to and following traumatic events predisposes individuals to PTSD, potentially by interfering with the consolidation of emotion-regulatory memory processes [56]. REM sleep fragmentation appears to be the key mechanism by which sleep disturbances contribute to impaired fear extinction and safety learning [64].

However, the specific mechanisms through which sleep-induced astrocytic dysfunction directly influences fear memory consolidation and extinction remain unclear. Future studies should investigate whether astrocytic gap junction disruption specifically affects fear-processing circuits in the amygdala and prefrontal cortex, and how compromised astrocytic energy metabolism impacts the synaptic plasticity required for fear extinction learning. Understanding these direct astrocyte-fear memory connections could reveal novel therapeutic targets that address sleep disturbances as modifiable risk factors for the development of PTSD and treatment resistance.

## 8. Conclusions

The emerging picture of astrocytic contributions to fear memory processing reveals that these cells are sophisticated information-processing units that actively participate in memory processing across the amygdala–hippocampus–prefrontal cortex network. Rather than simply supporting neurons, astrocytes serve as critical regulators of synaptic plasticity, neurotransmitter homeostasis, and circuit function during fear learning.

Key findings from recent research demonstrate that the following:

Amygdalar astrocytes are essential for fear memory acquisition and consolidation through gliotransmitter release, protein degradation, and modulation of amygdala–prefrontal cortex communication.

Hippocampal astrocytes regulate fear extinction through cholinergic-driven calcium dynamics and adenosine signaling.

Prefrontal cortex astrocytes contribute to fear memory regulation and extinction through glucocorticoid receptor signaling and structural plasticity.

Multiple molecular mechanisms underlie astrocyte–neuron communication, including gliotransmitter release, calcium signaling, regulation of protein synthesis, and epigenetic modifications.

Sex differences in astrocytic function have important implications in understanding and treating fear-related disorders.

Pathological alterations in astrocytic function contribute to fear-related psychiatric disorders and represent novel therapeutic targets.

The therapeutic implications of these findings are substantial, with the dysregulation of astrocytic GABA in the prefrontal cortex emerging as a key mechanism of impaired fear extinction and a promising therapeutic target. Multiple astrocytic pathways represent potential targets for the treatment of PTSD and related anxiety disorders. The development of astrocyte-targeted therapeutics may provide more effective and personalized treatments for these devastating conditions.

Future research should focus on understanding the temporal dynamics of astrocytic contributions to the different phases of fear memory processing, elucidating the molecular mechanisms underlying region-specific astrocyte functions, and developing astrocyte-targeted therapeutic interventions. The integration of advanced technologies with traditional behavioral and molecular approaches will continue to reveal new aspects of astrocytic function in fear memory processing.

As our understanding of astrocyte–neuron interactions continues to evolve, it has become increasingly clear that these cells are not merely passive participants but also active orchestrators of fear memory processing across the distributed neural network. This paradigm shift opens new avenues for understanding the fundamental mechanisms of learning and memory while providing hope for more effective treatments for fear-related psychiatric disorders.

## Figures and Tables

**Table 1:** Astrocytic Functions in Fear Memory by Brain Region.

Brain Region	Key Functions	Major Molecules/Pathways	Memory Phase	Experimental Evidence	Key References
Basolateral Amygdala (BLA)	Fear acquisition and consolidation; Amygdala-mPFC communication enhancement	Cx43 hemichannels (glutamate + D-serine release), EphrinB2-EAAT1 pathway, GFAP protein degradation, Astrocytic NMDA receptors (GluN2C)	Acquisition, Consolidation, Extinction	Chemogenetics (Gq-DREADD), Optogenetics (ChR2/ArchT), Spatial transcriptomics, Proteomic analysis	[8,9,10,18,19,20,21,22]
Hippocampus (CA1)	Contextual fear memory formation; Fear extinction regulation; Learning-associated astrocyte ensembles	Cholinergic signaling (α4/α7 nAChRs), ATP → Adenosine → A1 receptors, eIF2α protein synthesis regulation, NFIA transcription factor, PAPs local protein synthesis	Consolidation, Extinction, Recall	Optogenetics, Cholinergic modulation (donepezil), LAA ensemble manipulation, eIF2α genetic manipulation	[12,23,24,25,26,27]
Medial Prefrontal Cortex (mPFC)	Fear expression regulation; Extinction learning and recall; Remote memory maintenance; GABA-mediated tonic inhibition	Glucocorticoid receptors, Metabolic reprogramming (glucose/lipid metabolism), Structural plasticity, Sex-specific signaling, MAO-B-dependent GABA synthesis, GAT-3 mediated GABA release, Tonic GABA inhibition	Extinction, Recall, Remote memory	Chemogenetics (Gs-DREADD), Single-cell transcriptomics, GR knockout studies, Calcium imaging, Ultrasound stimulation	[13,28,29,30,31,32,33,34,35,36,37,38]

**Table 2:** Sex Differences in Astrocytic Fear Memory Processing.

Aspect	Males	Females	Functional Implications	Experimental Evidence	Reference
Protein degradation targets (Amygdala)	Cytoskeleton proteins, ATP synthesis enzymes, cell signaling molecules	Vesicle transport proteins, microtubule-associated proteins	Different molecular mechanisms underlying fear memory formation	Proteomic analysis following fear conditioning	[19]
Astrocytic calcium event characteristics	Lower amplitude and smaller size calcium events	Higher amplitude and larger size calcium events	Greater fear discrimination sensitivity and more profound impairments following astrocyte manipulation in females	Calcium imaging in BLA astrocytes	[40]
Glucocorticoid receptor function (mPFC)	No extinction memory deficit with GR knockout	Impaired extinction memory recall with astrocytic GR knockout	Sex-specific therapeutic targeting needed for PTSD treatment	Astrocyte-specific GR knockout studies	[13]
Stress response effects	Stress exposure reduces sex differences	Stress exposure eliminates natural calcium signature advantages	Stress vulnerability and resilience mechanisms differ between sexes	Early-life stress paradigms with calcium imaging	[40]
BDNF-pain relationship	Lower BDNF linked to pain only in depression	Lower BDNF in severe pain regardless of depression status	Sex-specific biomarker potential for fear-related disorders	Serum BDNF analysis in chronic pain patients	[44]
PTSD susceptibility	Lower overall prevalence	Higher prevalence and different symptom profiles	Requires sex-specific precision medicine approaches	Clinical and epidemiological studies	[13]

**Table 3:** Therapeutic Approaches and Clinical Evidence for Astrocyte-Targeted Fear Memory Interventions.

Category	Target/ Population	Intervention/ Biomarker	Mechanism/ Finding	Key Outcome	Reference
Therapeutic Targets	Fear/anxiety disorders	Donepezil	Acetylcholinesterase inhibition enhances astrocytic Ca^2+^ dynamics	Promotes fear extinction through α4/α7 nAChR activation	[23]
Therapeutic Targets	PTSD prevention	A1 receptor agonists	Mimic astrocyte-derived adenosine memory dampening	Selective consolidation interference without affecting acquisition/retrieval	[12]
Therapeutic Targets	PTSD	KDS2010	Reversible MAO-B inhibition restores astrocytic GABA homeostasis	Corrects astrocytic dysfunction underlying PTSD symptoms	[32]
Therapeutic Targets	Fear memory disorders	Cx43 modulators	Enhance gliotransmitter release (glutamate + D-serine)	Rescue memory deficits through NMDAR activation	[20]
Therapeutic Targets	Long-term memory deficits	EAAT1 enhancers	Improve astrocytic glutamate uptake	Selective enhancement of memory consolidation	[22]
Therapeutic Targets	PTSD, anxiety disorders	Psilocybin/MDMA	Reverse meningeal monocyte accumulation, restore astrocyte–neuron communication	Target peripheral immune-brain interactions via EGFR-PTPRS	[51]
Clinical Biomarkers	WTC Responders (*n* = 1520)	Plasma GFAP	Reduced levels in severe PTSD	Glial suppression rather than activation	[52]
Clinical Biomarkers	Post-9/11 Veterans (*n* = 550)	Plasma GFAP	Lower levels with blast exposure and psychological symptoms	Astrocytic dysfunction in trauma	[53]
Clinical Biomarkers	UK Military Personnel	Plasma GFAP	Elevated levels 8 years post-TBI	Poor functional outcomes, unemployment	[54]
Clinical Biomarkers	TBI Patients	GFAP, Tau	Higher levels in females with more severe symptoms	Sex-specific glial responses to trauma	[55]
Clinical Biomarkers	Chronic Pain Patients	Serum BDNF	Sex-specific patterns with pain severity	Potential biomarker for fear-related disorders	[44]
Precision Medicine	Fear conditioning	Protein degradation patterns	Different targets in males vs. females	Sex-specific molecular mechanisms	[19]
Precision Medicine	Extinction memory	Astrocytic glucocorticoid receptors	Female-specific extinction memory deficits with GR knockout	Hormonal interventions may be sex-dependent	[13]
Precision Medicine	Sleep disruption	Astrocytic markers (AQP4, Cx30, Cx43)	Reduced levels in sleep-deprived individuals	Sleep restoration as adjunctive therapy	[59]
Future Directions	Memory-specific astrocytes	LAA ensemble modulation	Selective targeting of learning-associated astrocyte populations	Precision targeting without global effects	[24]
Future Directions	Memory recall	NFIA transcription factor	Essential transcriptional regulator in astrocytes	Critical for memory recall function	[24]
Future Directions	Energy metabolism	Gap junction modulators	Restore astrocytic connectivity and energy support	Support synaptic plasticity and memory	[58]
Future Directions	Remote memory	Metabolic reprogramming	Glucose/lipid metabolism support in astrocytes	Long-term memory maintenance	[31]

## Data Availability

Not applicable, no new data were created.

## Abbreviations

AICP	(R)-2-amino-3-(4-(2-ethylphenyl)-1H-indole-2-carboxamido) propanoic acid
AMPA	α-Amino-3-hydroxy-5-methyl-4-isoxazolepropionic acid
ATP	Adenosine triphosphate
BDNF	Brain-derived neurotrophic factor
BLA	Basolateral amygdala
cAMP	Cyclic adenosine monophosphate
ChR2	Channelrhodopsin-2
CREB	cAMP response element-binding protein
Cx43	Connexin 43
DREADD	Designer receptors exclusively activated by designer drugs
EAAT1	Excitatory amino acid transporter 1
eIF2α	Eukaryotic initiation factor 2α
FGF2	Fibroblast growth factor 2
GABA	γ-aminobutyric acid
GFAP	Glial fibrillary acidic protein
GPCR	G-protein coupled receptor
GR	Glucocorticoid receptor
LAA	Learning-associated astrocyte
MAPK	Mitogen-activated protein kinase
MDD	Major depressive disorder
MDMA	3,4-methylenedioxymethamphetamine
mPFC	Medial prefrontal cortex
nAChR	Nicotinic acetylcholine receptor
NFIA	Nuclear factor IA
NMDA	N-methyl-D-aspartate
NMDAR	N-methyl-D-aspartate receptor
OGT	O-GlcNAc transferase
PAP	Perisynaptic astrocytic process
pBF	Posterior basal forebrain
PTSD	Post-traumatic stress disorder
REM	Rapid eye movement
TBI	Traumatic brain injury
